# Supplementary material for: The GenoDiabMar Registry: A Collaborative Research Platform of Type 2 Diabetes Patients
Source: J Clin Med. 2022 Mar 5;11(5):1431. doi: 10.3390/jcm11051431 (PMC8911424; doi:10.3390/jcm11051431)
Supplement: Supplementary file 1 [file jcm-11-01431-s001.zip › jcm-1541111-supplementary.pdf]

Journal of
Clinical Medicine

MDPI

Article

The GenoDiabMar Registry: A Collaborative Research Platform of Type 2 Diabetes Patients

Adriana Sierra [1], Sol Otero [2], Eva Rodríguez [1], Anna Faura [1], María Vera [1], Marta Riera [1], Vanesa Palau [1], Xavier Durán [3], Anna Costa-Garrido [3], Laia Sans [1], Eva Márquez [1], Vladimir Poposki [4], Josep Franch-Nadal [5,6], Xavier Mundet [5,7], Anna Oliveras [1], Marta Crespo [1], Julio Pascual [1,†] and Clara Barrios [1,*]

[1] Department of Nephrology, Hospital del Mar, Institut Hospital del Mar d'Investigacions Mèdiques, 08003 Barcelona, Spain; asierra@psmar.cat (A.S.); erodriguezg@psmar.cat (E.R.); afaura@psmar.cat (A.F.); mvera@psmar.cat (M.V.); mriera1@imim.es (M.R.); vpalau@imim.es (V.P.); lsans@psmar.cat (L.S.); eva.marquez.mosquera@psmar.cat (E.M.); aoliveras@psmar.cat (A.O.); mcrespo@psmar.cat (M.C.); julpascual@gmail.com (J.P.)
[2] Department of Nephrology, Consorci Sanitari Alt Penedès-Garraf . 08800 Barcelona, Spain; sospetita@hotmail.com
[3] Methodological and Biostatistical Advisory Service, Institut Hospital del Mar d'Investigacions Mèdiques, 08003 Barcelona, Spain; xduran@imim.es (X.D.); anna.costaga@e-campus.uab.cat (A.C.-G.)
[4] Department of Ophthalmology, Hospital del Mar, Institut Hospital del Mar d'Investigacions Mèdiques , 08003 Barcelona, Spain; vpoposki@psmar.cat
[5] Research Support Unit, University Institute for Research in Primary Care, Jordi Gol (IDIAP Jordi Gol), 08041 Barcelona, Spain; josep.franch@gmail.com (J.F.-N.); xmundet.bcn.ics@gencat.cat (X.M.)
[6] Biomedical Research Centre in Diabetes and Associated Metabolic Disorders (CIBERDEM), 28029 Barcelona, Spain
[7] Departamento de Medicina, Universidad Autonoma de Barcelona, 08193 Bellaterra, Spain
* Correspondence: cbarrios@psmar.cat or clarabarrios@hotmail.es; Tel.: +34-65-004-2149
† Current address: Department of Nephrology, Hospital Universitario 12 de Octubre, 28041 Madrid, Spain

Supplementary Methods

Supplementary Materials and Methods

Study design

The GenoDiabMar registry was designed as a prospective study and currently collected information regarding 650 Caucasian adults with T2D recruited from the nephrologist consultant of Hospital del Mar and six primary care centers from the Hospital del Mar health area, Litoral-Mar of Barcelona, Spain. The inclusion criteria were adults over 45 years old, diagnosed with T2D at least 10 years before the first study visit if there was no renal disease, and at any time, if renal damage was present. Information on the diagnosis of T2D was retrieved from the patient's electronic medical records. To avoid the inclusion of patients with pre-diabetes or impaired glucose tolerance and to ensure the T2D diagnosis, only patients under antidiabetic drugs were registered. Patients with autoimmune polycystic kidney disease or with previously known autoimmune diseases such as vasculitis or systemic lupus erythematosus were excluded.

Thus, patients who agreed to participate, met the inclusion criteria, and signed the informed consent were enrolled. Between February 2012 and July 2015, 650 T2D patients underwent a basal in-person medical visit (V1) performed by a nephrologist and a nurse. Medical history, demographics, physical examination, and laboratory data were registered along with the collection of blood and urine samples. In addition, an annual follow-up of all participants included at the baseline visit was performed to obtain complete analytical and clinical parameters, including new cardiovascular events, changes in the status of diabetic retinopathy and nephropathy, and mortality, by consulting participants′ electronic clinical reports. Between March 2017 and February 2020, living patients with functioning kidneys who did not require renal replacement therapy underwent the sec-

ond in-person visit (V2). Again, analytical, and clinical data, including changes in treatments, were registered. The second biological samples for biobank were collected in this second visit, performed on an average of 4.7 [0.65] years from the baseline visit.

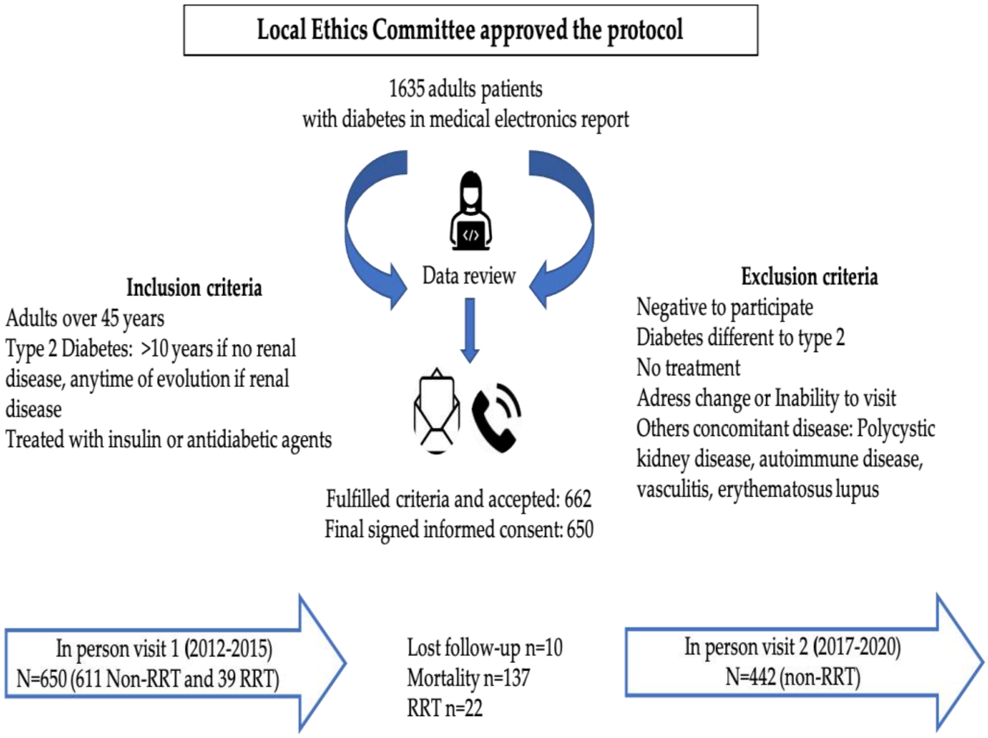

Figure S1. Flow-diagram of patient recruitment and follow-up. RRT: renal replacement therapy

The follow-up of the patients ended in February 2020, however, since they are patients from our health area from whom we have direct access, we could consult and log in their clinical and analytical variables if a study that required it were proposed. Contact can be made directly by email addressed to the principal Investigator of the project, Dr. Clara Barrios, or through the website of our research group on nephropathies of the Mar Institute of Medical Research (IMIM) (https://www.imim.es/programesrecerca/rct/en_nefropaties.html)

The study protocol was approved by the local Medical Ethics Committee of our research institute and the Steering Committee of the Primary Care Area. Patients were previously informed of the collection (of samples for the biobank and the use of their clinical data. National guidelines (Code of ethics of the professional association) and international guidelines (Declaration of Helsinki Fortaleza, Brazil, October 2013) were followed. Also, the confidentiality of the data was guaranteed in accordance with current regulations: Organic Law 3/2018, of December 5, Protection of Personal Data and guarantee of digital rights and Regulation (EU) No 2016/679 of the Parliament and Council of April 27, 2016, on Data Protection (RGPD). In addition, the study was carried out in accordance with the Biomedical Research Law (Law 14/2007). No binding data are shared for the patient and all data are pseudo-anonymized with a new coding of the medical record number and anonymous labeling of blood samples.

Data Registry.

Medical records and CV risk factors assessment.

Each participant completed a comprehensive questionnaire about their medical history, including information related to the presence and type of Diabetes Mellitus in the family history. Smoking status was registered as current smoker, previous smoker (smoke-free for more than 1 year), or non-smoker. Body mass index (BMI) was calculated as weight/height2 (Kg/m^2). The history of CV events was recorded at the baseline and annually and comprised, 1- ischemic heart disease (acute myocardial infarction, angina, cardiac revascularization); 2- cerebrovascular disease (cerebrovascular accident or transient ischemic attack) and 3- peripheral vascular disease (intermittent claudication, ischemic vascular ulcers, or surgical revascularization).

Hypertension was considered if the patient had previously been diagnosed or if they were under anti-hypertensive treatment. In addition, new cases of hypertension were identified if the patient presented systolic blood pressure (SBP) of 140 mmHg or higher, and/or diastolic blood pressure (DBP) of 90 mmHg or higher at the visit [1]. Blood pressure was measured with an automatically calibrated sphygmomanometer following a standardized protocol, recording the average of three measurements separated each by 3-5 min. Also, absolutes values were recorded in the dataset.

Dyslipidemia was considered both as quantitative variable including the absolute values of the lipid profiles component, i.e., determination of total cholesterol> 250mg/dL, LDL cholesterol> 130 mg/dL, HDL cholesterol <35/45 mg/dL (men/women respectively), triglycerides>200 mg/dL[2], and as qualitative variable if previously diagnosed by a physician or the patient used of lipid-lowering medication.

The presence or absence of diabetic retinopathy (DR) is recorded as a categorical variable and was diagnosed by fundoscopy performed by an ophthalmologist. It was classified as unknown retinopathy in the case of lack of fundoscopy. In addition, the presence of cataracts diagnosed in the ophthalmological examination was recorded.

The registry also gathered the medication in use at baseline and changes during the follow-up, including: Anti-hypertensive drugs distinguishing inhibitors of renin-angiotensin- aldosterone system, calcium antagonists, beta-blockers, diuretics, or combinations; Lipid-lowering treatment (statins, fibrates, or others such as omega3 polyunsaturated fatty acids or ezetimibe); antidiabetic drugs distinguishing insulin, all oral antidiabetic drugs, including tubular sodium-glucose co-transporter inhibitors (iSGLT2) or glucagon-like peptide-1 (GLP1-RA) receptor agonists.

Laboratory Data and sample management.

At baseline (V1) and at the last visits (V2), fasting venous blood and urine samples were collected. A 20 ml of EDTA (Ethylene diamine tetra acetic) blood sample was obtained from all participants. The samples were centrifuged (4000 rpm; 3 ml for 10 min at 4ºC) and stored at -80ºC until use. For participants undergoing renal replacement therapy with hemodialysis, fasting samples were taken before the mid-week dialysis treatment procedure. Serum, urine, DNA, and whole blood samples were stored in freezers of the Nephropathies Research Group (GREN) of the Institut Hospital del Mar d´Investigacions Mèdiques (IMIM) [3] and the Parc de Salut Mar Biobank (MARBiobanc) [4]. All samples for clinical analysis were centralized in a single laboratory, the Catalan Reference Laboratory (LRC). The main variables are summarized in tables 1 and 2.

Renal function was measured as estimated glomerular filtration rate (eGFR) from calibrated serum creatinine using the Chronic Kidney Disease Epidemiology Collaboration (CKD-EPI) equation [5]. Moderate albuminuria was defined as a urine albumin-to-creatinine ratio (ACR) of 30-299 mg/g, and severe albuminuria was defined as a urine ACR of 300 mg/g or greater. DKD was defined as: eGFR<60 ml/min/1.73m^2 and albuminuria >300 mg/g or albuminuria 30-299 mg/g and DR, regardless the eGFR. Patients were classified based on the degree of kidney disease following the KDIGO guidelines as grade 1-

2 if eGFR>90-60 ml/min/1.73m^2, grade 3 if eGFR; 59-30 ml/min/1.73m^2, grade 4 if eGFR; 29-15 ml/min/1.73m^2 and grade 5 if eGFR<15 ml/min/1.73m^2 [6].

Supplementary References

[1]: T. Unger *et al.*, "2020 International Society of Hypertension Global Hypertension Practice Guidelines," 6 May 2020https://doi.org/10.1161/HYPERTENSIONAHA.120.15026Hypertension. 2020;75:1334–1357.

[2]: F. Mach *et al.*, "2019 ESC/EAS Guidelines for the management of dyslipidaemias: Lipid modification to reduce cardiovascular risk," *European Heart Journal*, Volume 41, Issue 1, 1 January 2020, Pages 111–188, https://doi.org/10.1093/eurheartj/ehz455.

[3]: "Kidney Disease - IMIM Institut Hospital del Mar d'Investigacions Mèdiques.". Available online: https://www.imim.es/programesrecerca/rct/en_nefropaties.html. [Accessed: 20-Dec-2021].

[4]: "Parc de Salut Mar's biobank. Hospital del Mar Medical Research Institute, IMIM.". Available online: https://marbiobanc.imim.es/en_index/. [Accessed: 20-Dec-2021].

[5]: A. S. Levey *et al.*, "A new equation to estimate glomerular filtration rate," 2009 May 5;150(9):604-12. doi: 10.7326/0003-4819-150-9-200905050-00006.

[6]: Levin, A.; Stevens, P.E.; Bilous, R.W.; Coresh, J.; De Francisco, A.L.M.; De Jong, P.E.; Griffith, K.E.; Hemmelgarn, B.R.; Iseki, K.; Lamb, E.J.; et al. Kidney disease: Improving global outcomes (KDIGO) CKD work group. KDIGO 2012 clinical practice guideline for the evaluation and management of chronic kidney disease. *Kidney Int. Suppl.* **2013**, *3*A. Levin *et al.*, "Kidney disease: Improving global outcomes (KDIGO) CKD work group. KDIGO 2012 clinical practice guideline for the evaluation and management of chronic kidney disease," *Kidney International Supplements*. 2013.
